# Cell‐free DNA content in follicular fluid: A marker for the developmental ability of porcine oocytes

**DOI:** 10.1002/rmb2.12309

**Published:** 2019-12-21

**Authors:** Kana Ichikawa, Hidenori Shibahara, Komei Shirasuna, Takehito Kuwayama, Hisataka Iwata

**Affiliations:** ^1^ Department of Animal Science Tokyo University of Agriculture Kanagawa Japan

**Keywords:** blastocysts, cell‐free DNA, follicular fluid, mitochondria, oocytes

## Abstract

**Purpose:**

The present study examined the relationships among the amount of cell‐free‐DNA (cfDNA) in porcine follicular fluid (FF), the developmental ability of enclosed oocytes, and characteristics of granulosa cells and examined the effect of cfDNA content in maturation medium on the developmental ability of the oocytes.

**Methods:**

Oocytes and FF were collected from individual gilts, and the gilts were rated based on the ability of their oocytes to develop to the blastocyst stage and the amount of cfDNA in the FF. The copy numbers of mitochondrial DNA (Mt‐DNA) and nuclear DNA (N‐DNA) were measured by real‐time PCR and the DNA sequence. FF or cfDNA was added to the maturation medium, and the developmental ability of the oocytes was examined.

**Results:**

The amount of cfDNA was associated with apoptosis of the granulosa cells, and high‐cfDNA content in FF was associated with low developmental ability of oocytes. Supplementation of the maturation medium with FF containing high cf‐Mt‐DNA or with DNA extracted from the FF did not affect oocyte developmental competence.

**Conclusions:**

Cell‐free DNA content in FF is a marker for oocyte competence, but cfDNA in the oocyte maturation environment did not affect oocyte developmental ability.

## Graphical abstract

Cell‐free DNA contents in follicular fluid reflect low competent oocytes.

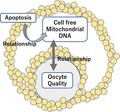

## 1 INTRODUCTION

Follicular fluid (FF) and granulosa cells (GCs) compose the sole environment for oocyte growth. FF comes from secretion by granulosa cells and circulation,^1^ and the characteristics of the FF are influenced by the physical condition of the donor and are a long‐term consequence of follicle development.^2^, ^3^

Noninvasive markers from FF are useful in predicting oocyte quality and accumulating clinical evidence points to a relationship between cell‐free DNA (cfDNA) content in FFs and the developmental competence of the enclosed oocytes. Women with a high pregnancy rate and high antral follicle count had low cfDNA content in their FF.^4^, ^5^ High fragmentation rate and low cleavage rate of day 3 embryos are associated with high‐cfDNA content in the corresponding FF.^6^ However, these results were obtained from clinical data sets that were not necessarily controlled for age, health, lifestyles, and eating habits.

The cfDNA present in the FFs varies in length from very small fragments to fragments over a thousand bp long and has two origins: nuclear or mitochondrial.^7^ A previous study showed that both cf‐N‐DNA and cf‐Mt‐DNA, analyzed by real‐time PCR, are closely related to cfDNA content (ng) in FF, and living granulosa cells actively secreted cf‐Mt‐DNA into the medium in response to mitochondrial dysfunction.^7^ However, other factors associated with this cfDNA in the follicles remain unclear. Furthermore, whether the amount of cf‐N‐ and/or cf‐Mt‐DNA reflects the developmental competence of oocytes remains unclear. For example, it has been reported that high developmental ability of oocytes is related to low cf‐Mt‐DNA content in FF, but not low cf‐N‐DNA.^8^ Another question that remains unanswered is whether the cfDNA content of FF is a causal factor for poor oocytes, or a consequence of poor follicle development. Guan et al^9^ reported that when DNA is added to the culture medium, it causes granulosa cell apoptosis. However, in that study, extensively high concentrations of DNA were directly added, and cfDNA is believed to be contained in extracellular vesicles or present and free floating.^7^, ^10^ Therefore, additional studies are needed to evaluate the significance of cfDNA in FF.

In the present study, we collected the ovaries of gilts which were kept in identical environments, and examined the relationships among characteristics of granulosa cells, developmental competence of oocytes, and the cf‐N‐ and cf‐Mt‐DNA contents in the corresponding FF. Furthermore, we investigated the effect of supplementation of maturation medium with FF containing high‐ or low‐cfDNA content, or with cfDNA extracted from the FF, on the ability of oocytes to develop to the blastocyst stage.

## 2 MATERIALS AND METHODS

### 2.1 Reagents and media

All reagents were purchased from Nacalai Tesque, unless otherwise stated. The medium used as IVM medium was porcine oocyte medium (POM)^11^ supplemented with 10% v/v porcine FF, 3 mg/mL polyvinyl alcohol, 0.5 mmol/L l‐cysteine, 10 ng/mL epidermal growth factor (Sigma‐Aldrich), 10 IU/mL equine chorionic gonadotropin (ASKA Pharma Co. Ltd), and 10 IU/mL human chorionic gonadotropin (Fuji Pharma Co. Ltd). FF was collected from the antrum follicles (3‐5 mm in diameter) of 100 gilts, centrifuged (10 000 × g for 5 minutes), and stored at −20°C until use. Porcine zygote medium 3 was used for in vitro culturing (IVC) of embryos.^12^ IVM was performed under atmospheric conditions of 5% CO₂ and 95% air at 38.5°C, and IVC was performed under atmospheric conditions of 5% O_2_, 5% CO_2_, and 90% N_2_ at 38.5°C.

### 2.2 Cumulus cell‐oocyte complex collection, IVM, activation, and IVC

Ovaries were collected from gilts obtained from a slaughterhouse and transferred to the laboratory within 30 minutes in phosphate‐buffered saline containing antibiotics at 37°C. Cumulus cell‐oocyte complexes (COCs) were collected from the antral follicles (AFs, 3‐6 mm in diameter) of ovaries and cultured in 100 µL droplets of IVM medium for 44 hours. After IVM, the oocytes were denuded from the surrounding GCs, parthenogenetically activated by a single electrical pulse of 60 V for 0.1 ms using a NEPA21 (NepaGene Co. Ltd), followed by incubation in PZM3 containing 10 µg/mL cytochalasin B and 10 µg/mL cycloheximide for 5 hours, and then cultured in PZM3 for 7 days to determine the rate of blastulation. The total cell number of the blastocysts was counted by Hoechst 33342 staining under a fluorescence microscope (Olympus).

### 2.3 DNA extraction from FF

Prior to DNA extraction, FF was diluted 400 times with water, and the diluted FF was mixed with an equal amount of DNA extraction buffer (Tris‐HCl, 20 mmol/L; Nonidet‐40; Tween 20, 0.9%; and proteinase K, 0.4 mg/mL). The buffer was then heated at 55°C for 30 minutes, followed by 98°C for 5 minutes, to obtain DNA. In preliminary experiments, it was confirmed that high concentrations of FF hamper the PCR, but the 800 times dilution of FF did not affect PCR efficiency.

### 2.4 Evaluation of cf‐N‐ and cf‐Mt‐DNA copy numbers in FF

The N‐DNA and Mt‐DNA copy numbers in FF were determined using real‐time PCR targeting the porcine mitochondrial genome and a single‐copy nuclear gene. PCR was performed using a CFX Connect™ real‐time PCR detection system (Bio‐Rad) with primers targeting a single‐copy gene or mitochondrial genome and Ssofast‐TM EvaGreen Supermix (Bio‐Rad). Primers used for detection of one‐copy gene were 5′‐agcagaatcaacaccatcggt‐3′ and 5′‐tggctccacccatagaatgc‐3′ (154 bp), and those for detection of mitochondrial genome were 5′‐atccaagcactatccatcacca‐3′ and 5′‐ccgatgattacgtgcaaccc‐3′ (155bp). Primers were designed using Primer3Plus (http://sourceforge.net/projects/primer3/) and the NCBI database (GCG glucagon, NC_010457.4 and *Sus scrofa* mitochondrion, complete genome NC_000845.1). PCR was performed with an initial denaturation at 95°C for 1 minutes, followed by 40 cycles at 98°C for 5 seconds and 60°C for 10 seconds. A standard curve was generated for each run using 10‐fold serial dilutions of the representative copies of the external standard. The external standard was the PCR product of the corresponding gene sequence, cloned into a vector using a Zero Blunt TOPO PCR cloning kit (Invitrogen). The PCR product was sequenced for confirmation prior to use. Amplification efficiencies of all assays were > 1.98.

### 2.5 Validation of measurement for cfDNA content in FF using DNA seq

Cell‐free DNA contained in FF has a wide variety of origins, and real‐time PCR targeting of one or two sequences in the nuclear and mitochondrial genome was validated by DNA seq. FF was collected from antral follicles (3‐6 mm in diameter) of 6 differential donor gilts. Cell‐free DNA extracted from FF was used for this analysis. Concentration and length distribution of cfDNA were evaluated by a Bioanalyzer (Agilent technologies) with the DNA 1000 kit (Agilent). Using 100 ng of cfDNA in each sample, sequence libraries were prepared with the KAPA HyperPrep Kit (KAPA Biosystems) according to the manufacture's protocol. Derived libraries were checked by the Bioanalyzer with a DNA 1000 kit and quantified with a KAPA Library Quantification kit (KAPA Biosystems). Diluted libraries of 10 nmol/L were sequenced on a HiSeq2500 (Illumina) as one lane of 100 bp paired‐end reads. Low‐quality data and adapter sequences were removed using CASAVA bcl2fastq (ver.2.18). Upon further filtering, low‐quality reads and ambiguous (N) bases were removed using CLC Genomics Workbench using the default settings. The remaining reads were aligned to the reference sequence Scrofa 11.1 (ftp://ftp.ensembl.org/pub/release-89/fasta/sus_scrofa/dna/) using CLC Genomics Workbench. All gene data have been registered (DRA006242; http://ddbj.nig.ac.jp/DRASearch/).

### 2.6 Preparation and rating of FF based on the developmental ability of enclosed oocytes

**Figure 1:**
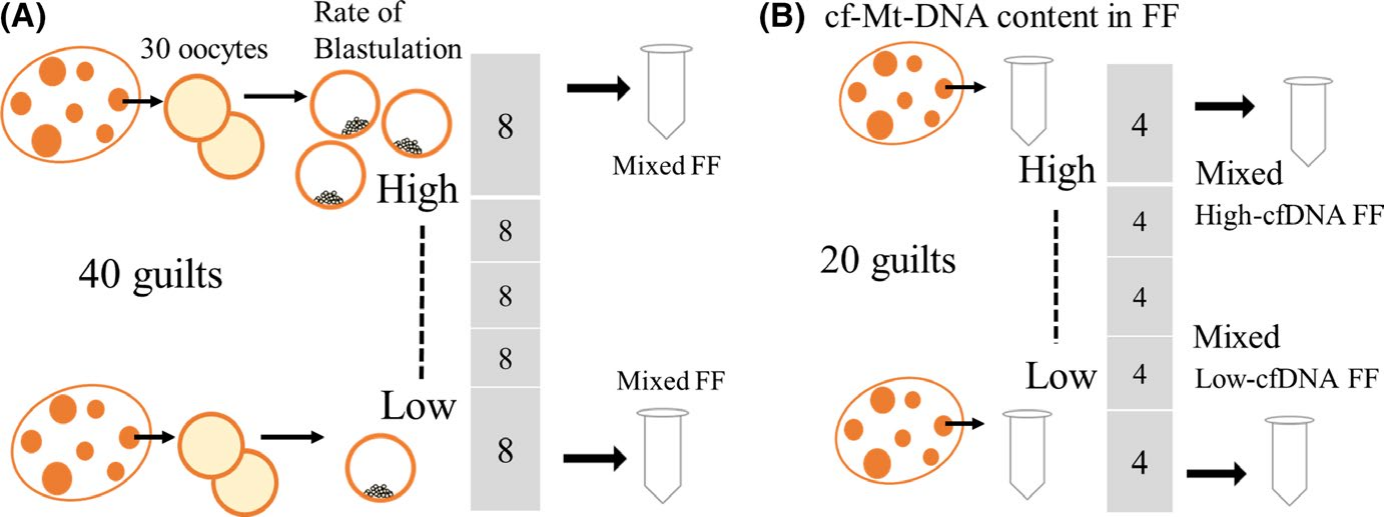
Preparation and rating of follicular fluid (FF) based on the developmental ability of oocytes (A) or on cell‐free mitochondrial (cf‐Mt‐) DNA content (B). A, Follicular contents were aspirated from antral follicles (3‐6mm in diameter) of 40 individual gilts. Thirty COCs were randomly selected and subjected to in vitro maturation and development following activation. The 40 gilts were rated based on the developmental rate of the corresponding oocytes to the blastocyst stage and were divided into 5 groups (from highest to lowest, with each group containing 8 gilts). FF from the highest and lowest groups was used to create High‐FF and Low‐FF. To obtain enough volume of FF for experiments, 2 randomly selected FFs were equally mixed, and 4 lots of FFs were prepared. B, FFs were collected from 20 gilts, and cf‐Mt‐DNA content in the FF was determined by real‐time PCR. The FFs were rated based on cf‐Mt‐DNA content and divided into 5 groups (from highest to lowest, each group containing 4 gilts). The top 4 and bottom 4 FFs were equally mixed to generate High‐cfDNA‐FF and Low‐cfDNA‐FFs. Six lots of High‐ and Low‐cfDNA‐FFs were prepared using a total of 120 gilts

A design of the preparation of FF rated on the developmental ability of oocytes is depicted in Figure 1A. Ovaries were collected from 40 gilts (a food lot of a farm), and follicular contents were aspirated from at least thirty AFs (3‐6mm in diameter) of each gilt ovary. COCs were extracted from the follicular content under a stereo microscope, after which the follicular content was centrifuged (3000 × g) for 10 minutes to obtain FF. Thirty randomly selected COCs were selected from each gilt and subjected to IVM followed by activation and subsequent IVC for 7 days, to determine the developmental ability of the oocytes. Based on the developmental ability of the oocytes, the corresponding FF was rated and divided into five categories: Highest (average developmental rate ± SEM, 34.0 ± 1.8%), High (21.4 ± 1.2%), Intermediate (11.4 ± 0.8%), Low (6.3 ± 0.5%), and Lowest (4.6 ± 0.1%), with each group comprised of 8 gilts. Using the FF from the Highest and Lowest groups, four High‐FF and Low‐FF batches were created (2 randomly selected FFs were equally mixed to obtain enough FF for supplementary experiments). In addition, the cf‐N‐ and cf‐Mt‐DNA contents in the High‐FF and Low‐FF were measured as described in Section 2.5.

### 2.7 Preparation and rating of FF based on cf‐Mt‐DNA content

FF was individually collected from the antral follicles of 20 gilts, rated based on their cf‐Mt‐DNA content, and then divided into 5 groups: Highest, High, Intermediate, Low, and Lowest, with each group comprised of 4 FFs (Figure 1B). The four FFs of either the highest or lowest groups were equally mixed to generate High‐cfDNA‐FF and Low‐cfDNA‐FF batches. We created 6 such batches of FF using 120 different gilts. These FFs were added to the IVM medium (10% or 30%). The average ± SEM of cf‐Mt‐DNA copy number in FF (1 µL) was 17 858 ± 1119 and 43 413 ± 7536 for Low‐ and High‐FF, respectively.

### 2.8 Preparation of cfDNA from FF to supplement the culture medium

FF was collected from the antral follicle (3‐6 mm in diameter) of 100 gilts, and the FF was subjected to DNA extraction. DNA content in the FF was extracted using a DNA Extraction Kit (MagMAX DNA Multi‐sample Ultra 2.0, Thermo Fisher). The DNA was diluted using water and added to the IVM medium at concentrations of 10 ng/μL, which is the concentration of DNA content in gilt FF (3‐6mm in diameter) as determined in previous reports.^7^

### 2.9 Comparison of cf‐N‐and cf‐Mt‐DNA contents in FF, with characteristics of the granulosa cells contained in corresponding follicles

**Figure 2:**
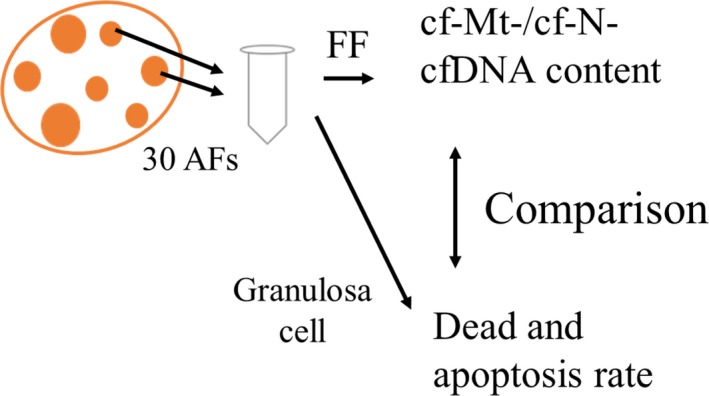
Comparison between amount of cf‐Mt‐DNA content in FF and characteristics of the granulosa cells. Follicular contents were obtained from 30 antral follicles (3‐6mm in diameter), and follicular fluid (FF) and granulosa cells were separated by centrifugation. DNA was extracted from the FF, and copy number of mitochondrial DNA in the FF was determined by real‐time PCR. Dead cell rate and apoptosis rate were determined by propidium iodide, Hoechst, and Annexin staining. Correlations between the characteristics of the granulosa cells and amount of cf‐Mt‐DNA in the FF were examined among 16 gilts

Follicle contents were aspirated from 30 randomly selected follicles (3‐6 mm in diameter) of ovaries of each gilt (N = 16) and centrifuged to separate FF and granulosa cells (200 g for 3 minutes) (Figure 2). The FF was further centrifuged at 3000 g for 10 minutes, and DNA was extracted from this FF (as described in 2.3) to determine copy numbers of cf‐N‐ and cf‐Mt‐DNA contents. Granulosa cells were washed three times and dispersed using disperse solution (Accumax, Innovative cell technologies) and stained with propidium iodide, Hoechst 33342, and Annexin V (Thermo Fisher). Two hundred cells from each sample were evaluated under a fluorescent microscope (LAS AF with Leica DMI 6000B, Wetzlar). The rate of dead cells (PI and Hoechst positive) and apoptotic cells (Annexin V and Hoechst positive but PI negative) was determined for each gilt. Correlations between the characteristics of the granulosa cells and cfDNA content in FF were examined among the 16 gilts.

### 2.10 Introduction of cfDNA into COCs or granulosa cells

Extracted DNA (see Section 2.3, final concentration 10 ng/μL) and Lipofectamine were used for the introduction, according to the protocol provided by the manufactures. Validation of the cfDNA introduction was determined by immunostaining against dsDNA. Granulosa cells were cultured on a glass chamber (Millicell, Merck Millipore) in 199 medium (Thermo Fisher) containing 5% FCS and the cfDNA‐Lipofectamine mix for 24 h, then subjected to immunostaining. Immunostaining was conducted as previously described.^13^ Antibodies used for the immunostaining were mouse anti‐dsDNA (1:200; ab27156) and goat anti‐mouse IgG Alexa Fluor 488 (1:500; Cell Signaling). Granulosa cells were observed under a fluorescence microscope (Leica DMI 6000B, Leica). COCs were incubated in IVM medium containing the cfDNA‐Lipofectamine mix for 44 h, and oocytes were activated to determine rate of development to the blastocyst stage.

### 2.11 Statistical analysis

Student's *t* test was used for analyzing the data of two groups, and the data (blastulation rate) from three or more groups were analyzed using analysis of variance (ANOVA), followed by Tukey's post hoc test. Percentages were arcsine transformed prior to analyses. Analyses were conducted using SPSS (ver. 21, IBM). *P* < .05 was considered significant.

## 3 RESULTS

### 3.1 Validation of cfDNA content in FF

**Figure 3:**
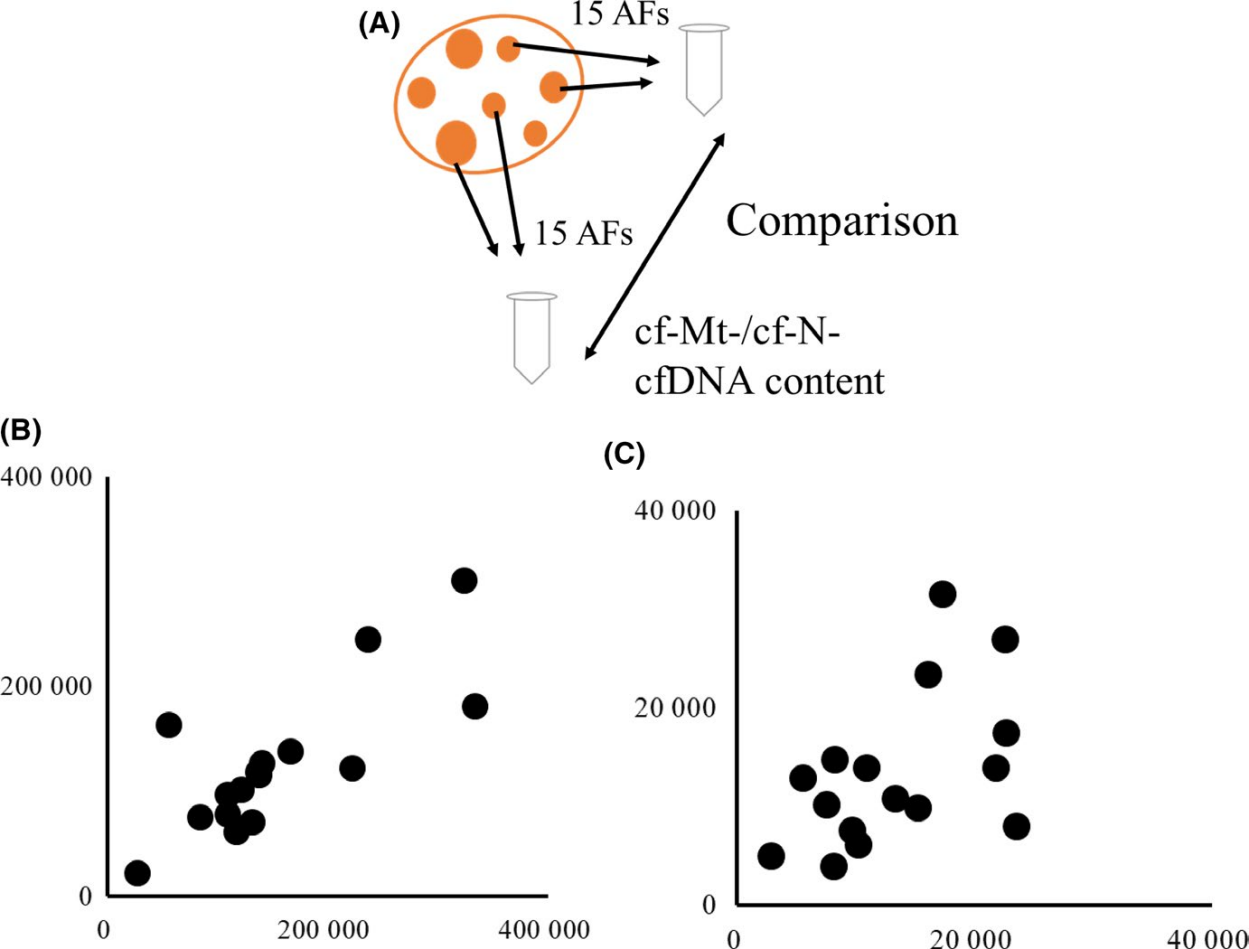
Copy number of cell‐free nuclear DNA (cf‐N‐DNA) and cell‐free mitochondrial DNA (cf‐Mt‐DNA) in FF. Two batches of follicular fluid were aspirated from 15 randomly selected antral follicles (3‐6 mm in diameter) of each of 16 gilts (A). cfDNA was extracted from the FF, and the cf‐N‐ and cf‐Mt‐DNA contents in FF were determined by real‐time PCR. The cf‐N‐DNA (B) and cf‐Mt‐DNA (C) were compared between the two FF batches. Y axis and X axis: cfDNA copy number in 1 µL of FF

Firstly, cf‐Mt‐ and cf‐N‐DNA contents were determined using 2 batches of FF, which were collected from each of 15 antral follicles (3‐5 mm in diameter) of individual gilts (Figure 3A). Significant correlation was found between the two cfDNAs among 16 gilts for cf‐Mt‐ (r = .78, *P* < .01; Figure 3B) and cf‐N‐DNA (r = .50, *P* < .05; Figure 3C). The results mean that the cfDNA content in FF determined using at least 15 AFs reflects the character of FF from each ovary. Therefore, the cfDNA content in FF from 30 AFs used in the following experiments reflects the character of the whole FF from each gilt.

From the DNAseq, read numbers mapped on the chromosomal and mitochondrial genomes are shown in Table 1. cfDNA found in FF comes from all chromosomes and the mitochondrial genome. Coverage depth (total read number mapped on each chromosomal gnome divided by total length of the chromosome) was about 11‐fold higher for the mitochondrial genome as compared to chromosomal. In addition, average coverage depth mapped on mitochondrial and nuclear sequences (155 and 154 bp) targeted by the real‐time primers used for the evaluation of mitochondrial and nuclear DNA copy number in FF (Section 2.4) was 15.12 ± 0.27 and 0.85 ± 0.02, respectively.

**Table 1:** Number of reads mapped on chromosome and mitochondria

Chromosomes	Length	No. of mapped read	Coverage depth
chr1	315 321 322	36 066 872	22.88
chr10	79 102 373	10 082 900	25.49
chr11	87 690 581	9 493 566	21.65
chr12	63 588 571	8 102 671	25.48
chr13	218 635 234	24 881 777	22.76
chr14	153 851 969	18 450 782	23.99
chr15	157 681 621	17 694 137	22.44
chr16	86 898 991	12 822 395	29.51
chr17	69 701 581	7 918 578	22.72
chr18	61 220 071	7 231 438	23.62
chr2	162 569 375	19 587 407	24.10
chr3	144 787 322	18 579 558	25.66
chr4	143 465 943	16 509 729	23.02
chr5	111 506 441	12 374 349	22.19
chr6	157 765 593	20 352 689	25.80
chr7	134 764 511	15 332 343	22.75
chr8	148 491 826	16 507 284	22.23
chr9	153 670 197	18 647 490	24.27
chrMT	16 613	21 881	263.42
chrX	144 288 218	16 378 812	22.70

Note

Six cell‐free DNA samples were analyzed, and total read number of the 6 samples were presented.

### 3.2 cfDNA content in FF relates to the apoptotic rate of corresponding granulosa cells

The next experiment addressed the causal factors of cfDNA secretion in FF. As described above (Section 2.9), the dead cell rate, apoptotic cell rate, and copy number of cfDNA in the FF were compared. As summarized in Table 2, the dead cell rate did not correlate with the rate of apoptosis in granulosa cells. In addition, the rate of dead cells correlated with neither cf‐N‐ nor cf‐Mt‐cfDNA copy number in the corresponding FF. On the contrary, the rate of apoptosis in granulosa cells was significantly correlated with the cf‐N‐DNA copy number in FF (r = .53, *P* < .05) and tended to correlate with the cf‐Mt‐DNA in FF (r = .44, *P* = .09).

**Table 2:** Correlations among dead cell and apoptosis rate of granulosa cells and cf‐N‐DNA and cf‐Mt‐DNA in FFs

r value	Dead	Apoptosis	cf‐N‐DNA	cf‐Mt‐DNA
Dead		−.42	−.21	.01
Apoptosis			.53*	.44
cf‐N‐DNA				.65^*^
cf‐Mt‐DNA				

Note

Granulosa cell condition and amount of cell‐free‐nucleic and mitochondrial DNA in follicular fluid (FF) were compared in 16 gilts.

*P < .05.

### 3.3 High developmental ability of oocytes is related to low cfDNA content in the corresponding FF

**Figure 4:**
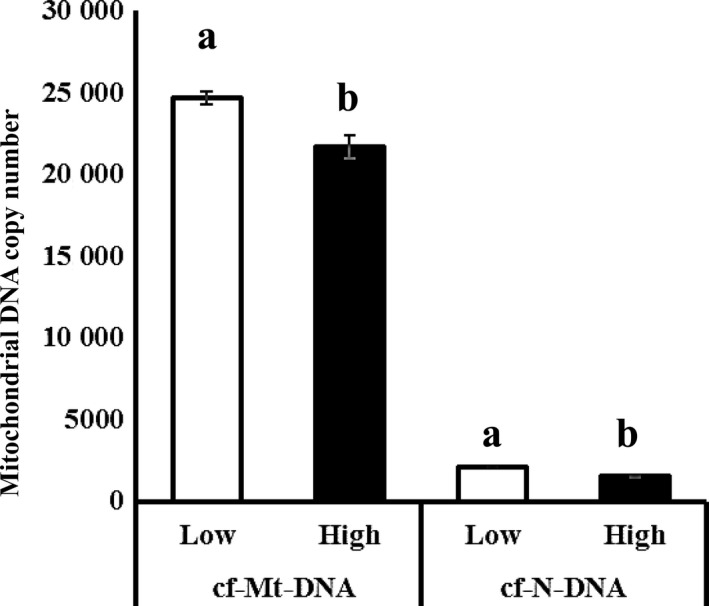
Comparison of copy number of cell‐free nuclear (cf‐N‐) and cell‐free mitochondrial (cf‐Mt‐) DNA between Low‐FF and High‐FF. Follicular fluid (FF) was collected from 40 gilts that were rated based on developmental ability of enclosed oocytes. The FF was rated based on the developmental rate to the blastocyst stage of enclosed oocytes. Cell‐free nucleic DNA (cf‐N‐DNA) and mitochondrial DNA (cf‐Mt‐DNA) between the High‐FF (N. 4) and Low‐FFs (N. 4); a‐b, *P* < .05. Bar represents SEM

Then, we prepared 4 High‐ and Low‐FF batches from 40 gilts based on the developmental ability of the enclosed oocytes (Section 2.6). Both cf‐N‐ and cf‐Mt‐DNA contents were higher for Low‐FF than for High‐FF (*P* < .05, Figure 4). To see whether the ability of FF to support oocyte development was linked to the developmental ability of enclosed oocytes, the Low‐FFs and High‐FFs were added to IVM medium at a concentration of 10%. This revealed that High‐FF significantly improved oocyte developmental ability to the blastocyst stage (30.0 ± 4.2 and 11.8 ± 3.0, *P* < .01; Table 3). The total cell number of blastocysts was similar between the two groups (High‐FF, 51.2 ± 3.3 vs Low‐FF, 54.4 ± 4.4).

**Table 3:** Effect of supplementation of IVM medium with High‐ or Low‐FF on developmental ability of oocytes

FF（10%）	No. of replicates	No. of oocytes	Rate of M2 (%)	No. of oocytes	Rate of blastulation (%)	TCM of blastocyst
High	4	160	89.0 ± 1.9	160	30.0 ± 4.2 b	51.2 ± 3.3
Low	4	160	81.0 ± 6.6	160	11.8 ± 3.0 a	54.4 ± 4.4

Note

COCs were cultured in IVM medium containing 10% of High‐ or Low‐FF. The maturation and blastulation rate, and the total cell number (TCM) of the blastocysts were measured. Data are represented as average ± SEM.

a,b: *P* < .05.

### 3.4 cfDNA content in the maturation medium did not affect oocyte developmental competence

**Figure 5:**
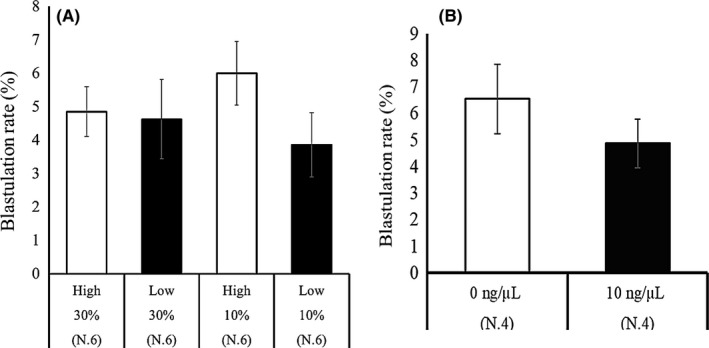
Effect of supplementation of maturation medium with High‐cfDNA‐FF and Low‐cfDNA‐FF on blastulation rate of the oocytes. A, Thirty COCs were cultured in IVM medium containing High‐cfDNA‐FF and Low‐cfDNA‐FF (10 or 30%), and developmental rate to the blastocyst stage was examined following activation. Experiments were repeated 6 times. B, Thirty COCs were cultured in IVM medium supplemented with 0 or 10 ng/μL of cfDNA extracted from FF. The oocytes were examined for their developmental rate to the blastocyst stage following activation. Experiment was repeated 4 times; Bar represents SEM

**Figure 6:**
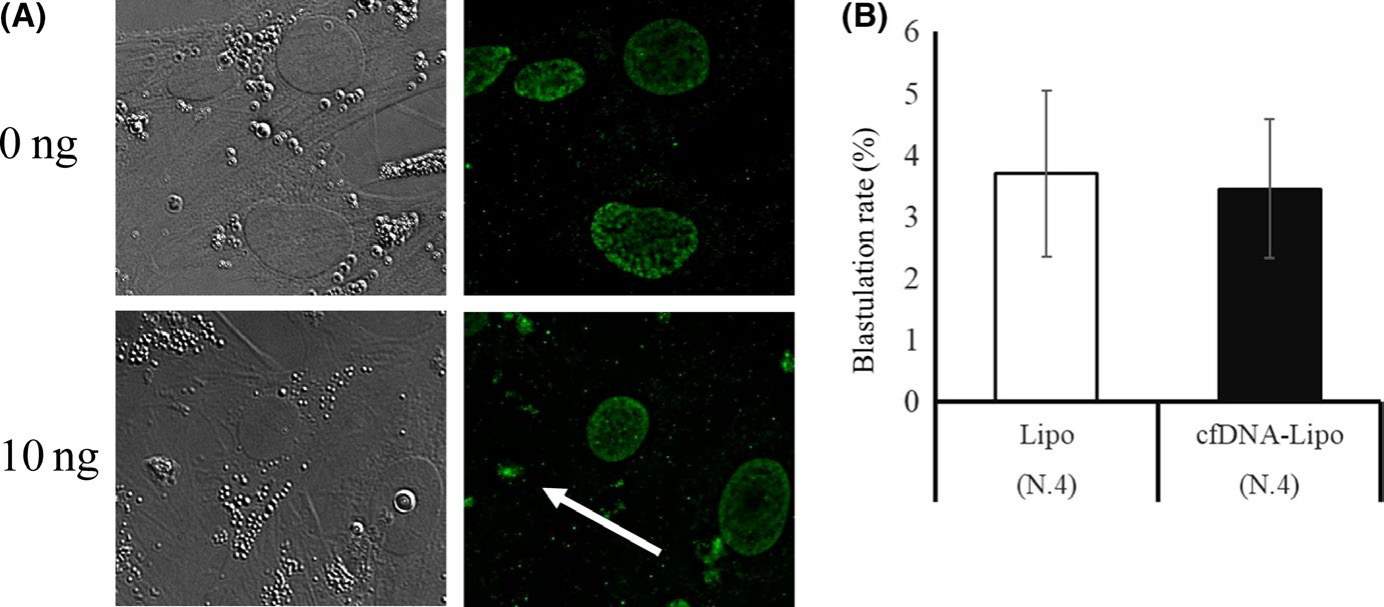
Effect of introducing cfDNA into COCs on the blastulation rate of the oocytes. A, Representative pictures of granulosa cells cultured with 0 or 10 ng/μL cfDNA extracted from FF for 24 hours followed by immunostaining against dsDNA. Cytoplasm of granulosa cells cultured with dsDNA had positive signals (arrow). B, Effect of coincubation of COCs with 0 or 10 ng/μL cfDNA extracted from FF on the rate of blastulation 7 days after activation. Bar represents SEM

We found that the origins of FF significantly affected oocyte development. We therefore addressed the question of whether cfDNA content in the maturation medium affects developmental ability to the blastocyst stage. FF containing high cfDNA or low cfDNA (Section 2.7, High‐cfDNA‐FF and Low‐cfDNA‐FF) was added to the maturation medium at a the concentration of 10 or 30%, and we found that neither the FF concentrations nor the cf‐Mt‐DNA content affected the rate of development of the oocytes to the blastocyst stage (High‐cfDNA‐FF 30%, 4.8 ± 0.7 vs Low‐cfDNA‐FF 30%, 4.6 ± 1.2, and High‐cfDNA‐FF 10%, 6.0 ± 1.0 vs Low‐cfDNA‐FF 10%, 3.9 ± 1.0; Figure 5A). Furthermore, when cfDNA was extracted from FF (Section 2.8) was added to the IVM medium at concentrations of 0 or 10 ng/μL, the developmental rate of the oocytes to the blastocyst stage was similar between the two DNA concentrations (Figure 5B). Moreover, we examined the effect of introducing cfDNA into the granulosa cells during in vitro maturation on oocyte developmental competence. As seen in Figure 6, treatment of cells with cfDNA and lipofection increased dsDNA content in the cytoplasm, but did not affect developmental ability of the oocytes to the blastocyst stage.

## 4 DISCUSSION

The present study showed that cfDNA is associated with apoptosis of the granulosa cells and that low cf‐N‐ and cf‐Mt‐DNA contents in FF are associated with high developmental ability of the corresponding oocytes. Furthermore, neither supplementation with FF containing high concentrations of cfDNA (as determined by cf‐Mt‐DNA) nor supplementation with cfDNA extracted from FF in the maturation medium adversely affected oocyte developmental competence.

Cell‐free‐DNA was evaluated by q‐PCR,^8^, ^14^ which targets one or two DNA sequences in the nuclear or mitochondrial genome. Prior to the use of real‐time PCR, comprehensive analysis of cfDNA in FF was needed to evaluate the presence of a target DNA sequence. The present DNA seq showed higher frequency of mitochondrial DNA compared with nuclear DNA. In addition, the DNA seq confirmed the presence of mitochondrial and nuclear DNA sequences targeted by the present real‐time PCR. The ratios of frequency of mitochondrial to nuclear DNA obtained by DNA seq were 11.0 for whole genome (Table 1) and 17.8 for real‐time PCR‐targeted sequence (0.85/15.12), respectively, and as obtained by the present q‐PCR was 10.4. From these, we can deduce that the present q‐PCR can precisely determine the frequency of cf‐N‐DNA and cf‐Mt‐DNA in FF.

Clinical data have shown that cf‐DNA in FF is a noninvasive marker of oocytes.^5^, ^6^, ^9^, ^15^ An in vitro model of the relationship between cfDNA in culture medium and competence of oocytes has been reported by Munakata et al,^16^ such that when oocyte‐granulosa cell complexes (OGCs) from early antral follicles were cultured in vitro, the quality of in vitro grown oocytes was associated with low cf‐Mt‐DNA content in the spent culture medium. The present study is the first to confirm that both cf‐N‐ and cf‐Mt‐cfDNA contents in FF were low for follicles containing competent oocytes using young, large animals kept in similar conditions.

Cell‐free‐DNA is reported to be derived from apoptotic granulosa cells,^9^ but others suggest that cfDNA does not reflect apoptosis or necrosis of cells.^16^ The present study compared cf‐N‐ and cf‐Mt‐DNA contents in FF with the apoptosis rate of corresponding granulosa cells and found a significant relationship for cf‐N (*P* < .05) and trend for cf‐Mt‐DNA (*P* = .09). Contrary to the apoptosis rate, the dead cell rate of the granulosa cells did not relate to either cfDNA in FF. This suggests that cfDNA content reflects certain ongoing cellular dysfunctions (apoptotic cells) in follicles, but not accumulated events in FF (dead cells).

In in vitro studies, it has been reported that culture conditions and culture substrates affect the release of cfDNA.^16^, ^17^ In addition, differential molecular backgrounds underlying the secretion of cf‐N‐ and cf‐Mt‐DNA have been reported. Munakata et al^16^ showed that cf‐N‐DNA content in the spent culture medium more closely, and negatively, relates to the survival rate of granulosa cells compared with cf‐Mt‐DNA content. Furthermore, mitochondrial membrane uncoupler (CCCP)‐induced mitochondrial dysfunction increased cf‐Mt‐DNA in the spent culture medium of porcine granulosa cells, but not cf‐N‐DNA.^7^ Moreover, treatment of granulosa cells with inhibitors of proteasomes or autophagy decreased or increased cf‐Mt‐DNA in the culture medium, respectively.^18^ In the present study, we found a significant correlation between cf‐N‐ and cf‐Mt‐DNA contents in FF (r = .544, *P* < .01), while a significant relation was found for apoptosis rate of granulosa cells and cf‐N‐DNA but not for cf‐Mt‐DNA. The results suggest differential molecular backgrounds might be included in the secretion of the two cfDNAs.

Interestingly, in the present study, we found that FF collected from ovaries containing highly developmentally competent oocytes (High‐FFs) had an increased ability to support oocyte maturation, and subsequent embryonic development, compared with Low‐FF (FF collected from ovaries having poorly developmentally competent oocytes). These results remind us that FF itself decides the oocyte quality, and certain adverse factors such as high cfDNA content in Low‐FF may aggravate the oocyte quality. Mitochondrial DNA has been reported to be a causal factor for cellular stress and apoptosis.^19^ Induction of mitochondrial dysfunction such as by CCCP treatment increased cytoplasmic DNA content, which induced cellular inflammation.^20^ Therefore, it is hypothesized that high‐cfDNA content induces high cellular stress, which impairs oocyte competence. In testing this hypothesis, Kostyuk et al 21 have reported that cfDNA induced ROS generation in human mesenchymal stem cells and addition of cfDNA to the culture medium induced apoptosis of human granulosa cells.^9^ However, in that study, 4 mg/mL cfDNA was added to the culture medium, whereas the cfDNA content in porcine FF is roughly 10 µg/mL.^7^ Furthermore, in the FF and plasma, cfDNA is contained in exosomes and extracellular vesicles,^6^, ^10^ and addition of DNA directly to the medium does not reflect the follicular environment. In this context, the present study addressed this hypothesis using two methods: addition of FF containing high cfDNA and low cfDNA to the IVM medium and addition of cfDNA extracted from FF to the IVM medium. When High‐cfDNA‐FF and low‐cfDNA‐FF were added to IVM medium at a concentration of 10%‐30%, no difference was found in the developmental ability of the oocytes. In addition, supplementation of maturation medium with cfDNA extracted from the FF (10 ng/μL) did not affect oocyte developmental competence. These results suggest that the cfDNA content did not impair oocyte maturation or subsequent developmental ability to the blastocyst stages. In this study, we further introduced cfDNA into the granulosa cells using Lipofectamine and found that an increase in cfDNA content in granulosa cells from culturing granulosa cells with cfDNA and Lipofectamine increased cfDNA fragments in the cytoplasm. However, this introduction of cfDNA into the cells did not affect oocyte competence. It is important to note that the oocyte quality of gilts changes drastically between seasons and at different farms, which affects the developmental abilities of oocytes and may mask the intrinsic effects of cfDNA. In addition, our experiment used cfDNA within a range previously found to be normal in pig FF (8‐12 ng/μL).^7^ Highly oxidized DNA and high concentration of DNA are reported to induce cell death process 21; therefore, the threshold of concentrations and types of cfDNA which adversely affect oocyte quality remains to be examined.

In conclusion, cfDNA content in FF reflects the developmental ability of enclosed oocytes and apoptotic conditions of the granulosa cells. The presence of cfDNA in FF and culture conditions did not adversely affect the developmental ability of the oocytes to the blastocyst stage when the concentration of the cfDNA fell within normal physiological ranges.

## CONFLICT OF INTEREST

Kana Ichikawa, Hidenori Shibahara, Komei Shirasuna, Takehito Kuwayama, and Hisataka Iwata declare that they have no conflict of interest.

## HUMAN RIGHTS STATEMENTS AND INFORMED CONSENT

This article does not contain any studies with human subjects.

## ANIMAL STUDY

In this study, porcine ovaries were collected from a slaughterhouse. The ovaries were discarded without any use for edible meat, and thus, this study was approved by the Ethical Committee for Animal Experiment of Tokyo University of Agriculture. All institutional and national guidelines for the care and use of laboratory animals were followed.
